# Endurance Training Alleviates Metabolic‐Associated Fatty‐Liver Disease (MAFLD)‐Related Testicular Impairments via Endoplasmic Reticulum Stress Regulation

**DOI:** 10.1002/jcla.70042

**Published:** 2025-05-01

**Authors:** Nastaran Teimouri, Vahid Kazemizadeh

**Affiliations:** ^1^ Clinical Research Development Center Imam Reza Hospital, Kermanshah University of Medical Sciences Kermanshah Iran; ^2^ Cardiovascular Research Center Health Policy and Promotion Institute, Kermanshah University of Medical Sciences Kermanshah Iran; ^3^ Department of Sport Physiology Faculty of Sport Sciences, Razi University Kermanshah Iran

**Keywords:** endoplasmic reticulum stress, fatty liver, male infertility, metabolic syndrome, physical activity

## Abstract

**Background:**

Metabolic‐associated fatty liver disease (MAFLD), the most prevalent liver disorder globally, affects 20%–40% of the population and presents significant health challenges. Studies link MAFLD to male reproductive dysfunction, highlighting the need for effective interventions. This study investigates the impact of MAFLD on testicular function and evaluates the protective role of endurance training, with a focus on the GRP78‐IRE‐1α‐ATF6 signaling pathway.

**Methods:**

Forty‐four rats were allocated into two dietary groups (*n* = 22 each): a standard diet control group (C) and a high‐fat diet supplemented with fructose water group (FL). After 17 weeks, histological analysis confirmed MAFLD development in the FL group, while the control group showed no pathological changes. Each dietary group was further subdivided into sedentary and endurance‐trained (T) subgroups (*n* = 10 per subgroup), resulting in four experimental groups: C, C + T, FL + T, and FL. At the end of the research, thyroid stimulating hormone (TSH), sex hormones (testosterone), tumor necrosis factor‐alpha (TNF‐a) as well as GRP78, IRE‐1α, and AFT6 expression were assessed.

**Results:**

Our results indicated that MAFLD led to significant weight gain, disrupted serum levels of thyroid‐stimulating hormone, and impaired sex hormone profile. Additionally, MAFLD triggered ER stress, evidenced by dysregulated expression of genes in the GRP78‐IRE‐1α‐ATF6 pathway. Remarkably, endurance training mitigated these adverse effects by normalizing hormonal profiles and restoring the expression of ER stress‐related genes. These findings highlight the critical role of ER stress in MAFLD‐induced male reproductive dysfunction.

**Conclusion:**

Overall, the present study suggests endurance training as a promising treatment strategy for addressing MAFLD and its associated reproductive complications.

## Graphical abstract

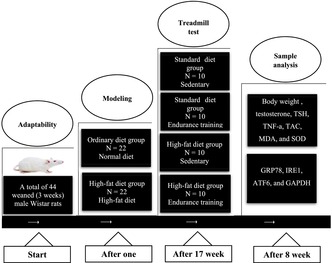

## 1 Introduction

Metabolic‐associated fatty‐liver disease (MAFLD) is the most prevalent liver disorder, affecting 20%–40% of the global population. Its incidence has risen sharply in recent years, driven primarily by the growing obesity epidemic in modern societies [1]. Among the various factors contributing to MAFLD, hypothyroidism has emerged as a potential contributor, although its exact role remains poorly understood due to the complex pathogenesis of the disease [2]. Thyroid hormones regulate essential physiological processes in multiple organs, with the liver being a key target [3]. Impaired thyroid function can lead to hypercholesterolemia, a fundamental factor in the development of hypothyroidism‐induced MAFLD [4]. MAFLD is strongly linked to several extrahepatic conditions, including endocrine disorders such as diabetes, metabolic syndrome, thyroid dysfunction, and insulin resistance, as well as cardiovascular and chronic kidney diseases [4, 5, 6]. Furthermore, researchers have associated MAFLD with male reproductive health issues, including erectile dysfunction, hypogonadism, and infertility, often as part of its connection with metabolic syndrome [7, 8].

Infertility is a reproductive system disease that occurs when a couple is unable to conceive after a year or more of regular, unprotected sex [9]. This condition impacts approximately 180 million couples worldwide [10]. Male infertility contributes to nearly half of all cases where couples face difficulty conceiving, with endocrine disorders such as hypothyroidism playing a significant role [11, 12]. MAFLD is a hallmark of metabolic syndrome, which is strongly associated with male reproductive and sexual problems. Recent studies also link MAFLD to poor semen quality and an increased risk of testosterone deficiency in men [7]. Low testosterone levels, in turn, contribute to diminished nocturnal and morning erections, reduced ejaculate volume, delayed ejaculation, disturbed spermatogenesis and reproductive function, and ultimately, infertility [13].

Thyroid hormones play a critical role in the relationship between MAFLD and metabolic dysfunction [14]. Disorders of thyroid function, particularly hypothyroidism, significantly increase the risk of developing MAFLD [15]. This connection is attributed to the influence of thyroid hormones on cholesterol and fatty acid metabolism within the liver [16]. Even subclinical hypothyroidism has been identified as an independent risk factor for MAFLD [17, 18]. Interestingly, research suggests that the association between thyroid hormone levels and MAFLD may vary depending on the specific subtype of the disease, with obesity and metabolic disorders serving as mediating factors [17]. However, this relationship is complex and may not follow a straightforward linear pattern, leading to some controversy in the findings. While hypothyroidism is not common in men, it can adversely affect sperm quality and alter the physical properties of seminal fluid, further highlighting its impact on male reproductive health [19].

Endoplasmic reticulum (ER) stress arises when cells fail to properly fold and process proteins within the ER, a condition triggered by genetic predispositions or environmental factors [20]. This cellular dysfunction has been implicated in various health conditions, including neurodegenerative disorders, diabetes, and cancer [21, 22]. Notably, ER stress plays a pivotal role in the onset and progression of MAFLD [23]. Similarly, testicular dysfunction, caused by factors such as varicocele, obesity, diabetes, aging, inflammation, or environmental and lifestyle influences, is often associated with an accumulation of unfolded or misfolded proteins, reflecting compromised ER function [24]. A key component of the ER stress response is the glucose‐regulated protein 78 (GRP78), inositol‐requiring enzyme 1 α (IRE‐1α), and activating transcription factor 6 (ATF6) signaling pathway, which governs cellular processes like apoptosis and autophagy [25]. This pathway has been directly linked to male infertility, as ER stress disrupts spermatogenesis and compromises the integrity of the blood‐testis barrier (BTB), further emphasizing its significance in reproductive health [26, 27].

Recent studies suggest that regular moderate‐intensity exercise can attenuate ER stress by modulating gene and protein responses, offering protective effects against metabolic and reproductive dysfunctions [28]. Exercise is known to improve male fertility under conditions such as obesity and diabetes [29]. Aerobic exercise, in particular, has demonstrated benefits in reducing insulin resistance, improving mitochondrial function, enhancing fatty acid metabolism, and mitigating inflammatory processes [30, 31, 32, 33]. However, the benefits are dose‐dependent, with high‐intensity exercise potentially exacerbating stress responses if performed without adequate rest [34, 35].

While significant research has examined the effect of exercise on ER stress across various tissues, including brain [36, 37], heart [38], muscle [39], and diseases like type 2 diabetes [40, 41], Parkinson's [42, 43], and cancer [44], the role of ER stress in MAFLD‐induced male reproductive dysfunction remains unexplored. Specifically, the potential of endurance training to mitigate MAFLD‐induced male reproductive issues via modulation of the GRP78‐IRE‐1α‐ATF6 pathway in testicular tissue has not been investigated. Hence, the present study aims to bridge the knowledge gap by investigating the effect of endurance training on MAFLD‐induced male sexual and fertility impairments, with a specific focus on the GRP78‐IRE‐1α‐ATF6 signaling pathway in testicular tissue.

## 2 Materials and Methods

### 2.1 Animals

A total of 44 weaned male Wistar rats were purchased from the Pasteur Institute (Tehran, Iran). The animals were held under standard conditions, including a 12‐h dark/12‐h light period, 23°C ± 1°C temperature, and 50%–60% humidity, and had free access to water and food throughout the study. All procedures were performed under the guidelines established by the Animal Care and Research Ethics Committee of Kermanshah University of Medical Sciences (IR.KUMS.AEC.1402.047). All methods were carried out in accordance with ARRIVE guidelines and regulations (https://arriveguidelines.org).

### 2.2 Experimental Design

**FIGURE 1:**
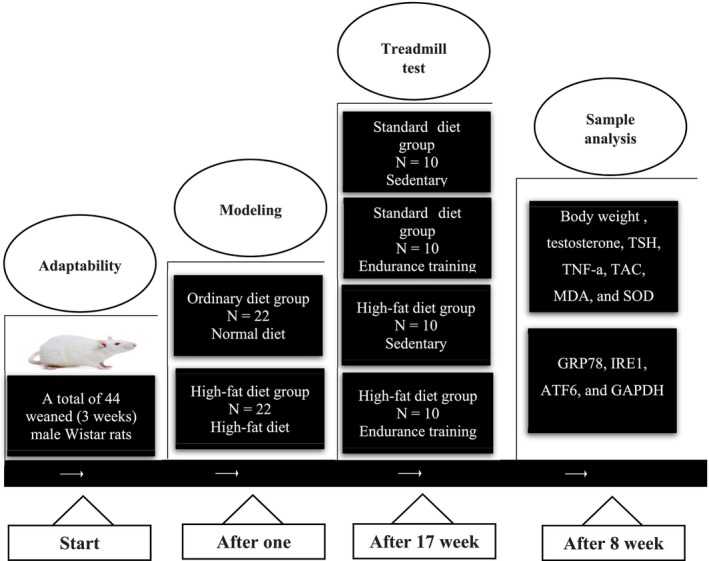
The schematic experimental design of the study. Rats were randomly assigned to a standard diet or a high‐fat diet group. After 17 weeks, two rats from each group were sacrificed for liver tissue collection and H&E staining to confirm MAFLD. Subsequently, each dietary group was divided into sedentary and endurance‐trained subgroups, forming four experimental groups.

The schematic design of the experimental protocol is presented in Figure 1. The rats had unrestricted access to conventional laboratory food and piped water, and they were acclimatized to the environment for one week before the commencement of the experiment. In the following phase, 22 rats were randomly assigned to the standard diet group and 22 to the high‐fat diet group, including 60% fat and high‐fructose, including 25% fructose with free access (Shafa Arad Daru, Iran) (Table 1).

**TABLE 1:** Animal feed characteristics.

Macronutrient	Regular	High‐fat diet
Protein (% wt)	23	24
Carbohydrate (% wt)	50	26
Fat (% wt)	51	35
Fat (% total c)	12	60
Calories (c/g)	3_/_1	5_/_2

After 17 weeks, two rats from each group were sacrificed, and Liver tissue was collected for hematoxylin–eosin (H&E) analysis to confirm the presence of MAFLD in the high‐fat diet + high‐fructose group and the absence of disease in the control group. The remaining 20 rats in the standard diet group were divided into two groups: control (C), endurance training (T), and 20 rats in the high‐fat diet group, along with 25% fructose water, were divided into two groups: fatty liver (FL) and fatty liver + endurance training (FL + T) (each group containing ten rats).

According to Rodrigues et al. [45], the T‐groups completed an 8‐week exercise intervention. It should be noted that there was no difference in food intake of the standard group vs. the High‐fat diet group rats before and after starting the endurance exercise regime. At the conclusion of the study, following blood sample collection, each anesthetized animal was placed in a sealed chamber filled with isoflurane to ensure humane euthanasia. Subsequently, a complete hepatectomy was performed. Testicular tissues were then promptly harvested, weighed, rinsed with saline solution, and stored at −80°C to preserve their integrity for subsequent analysis [46].

### 2.3 Endurance Training Protocol

An endurance training protocol was implemented for eight weeks after confirmation of MAFLD in the experimental rats. The endurance training protocol program for this investigation is shown in Table 2. All training sessions occurred after the animals' sleep cycle and between 9 and 11 A.M. [6].

**TABLE 2:** Endurance training program protocol in different weeks.

Week	Time (min)	Speed (m/min)	Slope (%)
First	15	10	5
Second	15	10	5
Third	20	15	5
Fourth	20	15	5
Fifth	30	20	5
Sixth	30	20	5
Seventh	40	25	5
Eighth	40	25	5

### 2.4 Hormones Assessment

48 h after the last exercise session (or at the corresponding time for non‐exercised animals), and after 12 h of fasting animals were anesthetized via intraperitoneal injection of a combination of xylazine (5 mg/kg) and ketamine (60 mg/kg). To analyze blood variables, a blood sample was obtained from the sedated rats' right atrium before testicle removal. For measuring serum thyroid‐stimulating hormone (TSH), testosterone, and tumor necrosis factor‐alpha (TNF‐a) levels, samples were centrifuged at a speed of three thousand revolutions per minute. After separating, the serum was stored at −80°C. To measure the values of serum thyroid‐stimulating hormone, testosterone, and TNF‐a levels by the ELISA method, commercial animal model kits (Moonblind Inc., USA) were used according to the instructions provided by the manufacturer.

### 2.5 Testicular Oxidative Stress

Testicle tissue (200 mg) taken from the same part of the testicle was tested for testicle oxidative stress. Testicle malondialdehyde (MDA), superoxide dismutase (SOD) activity, and total antioxidant capacity (T‐AOC) levels were quantified according to the manufacturer's protocol. The results were corrected for protein content [47].

### 2.6 Quantitative Real‐Time Polymerase Chain Reaction (PCR) Analysis

Total RNA was extracted with the RNX‐Plus Solution Kit (Sinaclone, Iran). The quality and quantity of the extracted RNA were controlled by the UV/Visible Spectrophotometer (Jenway, Malaysia). Regarding the concentration of the extracted RNA and the maximum capacity of the cDNA synthesis kit (Sinaclone, Iran), the extracted RNA was applied for cDNA synthesis immediately. The quantitative real‐time PCR was performed by a real‐time thermal cycler Rotor‐Gene Q (Qiagen, Germany) and the SYBR Green Master Mix (Ampliqon, Denmark). The thermal cycle protocol used in real‐time PCR included a temperature of 95°C for 10 min followed by 40 cycles of 15 s at 95°C, 20 s at 60°C, and 30 s at 72°C. The sequences of all the genes are listed in Table 3, and glyceraldehyde 3‐phosphate dehydrogenase (GAPDH) was used as a housekeeping gene.

**TABLE 3:** Sequences of the primer pairs used for RT‐PCR.

Primer list				
No.	Gene	Forward sequence	Reverse sequence	Ref
1	GRP78	5′‐GCCAACTGTAACAATCAAGGTCT −3′	5′‐TCAGGTGTCAGGCGGTTTT −3′	[48]
2	IRE‐1α	5′‐TTGACTATGCAGCCTCACTTC‐3′	5′‐AGTTACCACCAGTCCATCGC‐3′	[49]
3	AFT6	5′‐TATCCCTCCACCTCCATGTCA −3′	5′‐TCTCGATTTGGTCCTTTCCACT‐3′	[50]
4	GAPDH	5′‐CTCCCATTCTTCCACCTTTG‐3′	5′‐CTTGCTCTCAGTATCCTTGC‐3′	[49]

### 2.7 Statistical Analysis

The Shapiro–Wilk test was utilized to examine the data's normal distribution. The results of the Shapiro–Wilk test showed that the distribution of all the variables in the research was normally distributed, so parametric tests were used to perform statistical calculations. The one‐way analysis of variance statistical method and Tukey's post hoc test were considered at a significance level of *p* = 0.05 to reject or accept hypotheses, while the independent t‐test was utilized to evaluate differences between two groups. Pearson correlation assessed bivariate correlations between changes in all variables. All statistical calculations were performed using SPSS software version 26 (IBM Corporation, Armonk, NY) for data analysis and interpretation.

## 3 Results

### 3.1 Endurance Training Mitigates Weight Gain and Obesity Induced by High‐Fat Diet

**FIGURE 2. Analysis of body weight of the studied rats:**
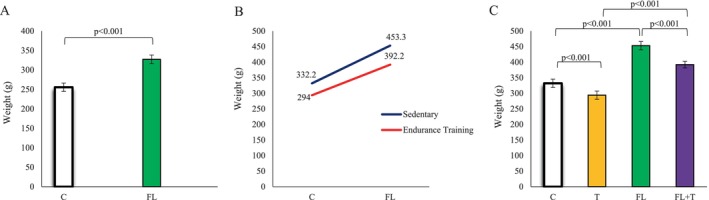
Weekly changes in body weight of rats during modeling (Figure 3A), and after 8 weeks of endurance training (Figure 3B,C). Data are presented as means ± standard deviations (average of 3 trials). C, control; FL + T, Fatty liver + endurance training; FL, fatty liver; T, endurance training.

Our results revealed that after 17 weeks of exposure to a high‐fat diet combined with fructose water, the FL group exhibited a significantly greater body weight gain compared to the control (C) group (*p* < 0.001) (Figure 2A). Also, statistical analysis showed that endurance training significantly decreased body weight level (*p* < 0.001), but HFD increased body weight level (*p* < 0.001). There was a statistically significant interaction between the effects of endurance training and HFD on body weight levels (*p* < 0.001) (Figure 2B). Additionally, following eight weeks of endurance training, notable differences in body weight were observed among the four experimental groups. Endurance training effectively reduced body weight in rats within the training group compared to the control group. Similarly, a significant reduction in body weight was recorded in the FL + T group compared to the FL group (*p* < 0.001) (Figure 2C).

**FIGURE 3. Analysis of serum testosterone, TSH, and TNF‐α levels:**
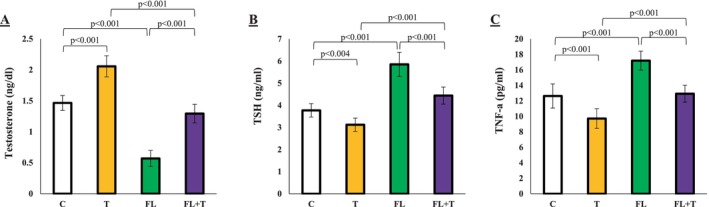
The effect of MAFLD with or without endurance training on testosterone, TSH, and TNF‐a levels in the controls and experimental groups. Data are presented as mean ± standard deviation (average of 3 trials). C, control; FL + T, Fatty liver + endurance training; FL, fatty liver; T, endurance training.

On the other hand, the present study showed that inactivity significantly increased the weight of rats in group C and group FL, but endurance training prevented weight gain in group T and group FL + T. While exercising reduces the complications caused by consuming a high‐fat diet, the best treatment option for people with MAFLD is lifestyle and diet modification along with exercise.

### 3.2 Endurance Exercise Restores Testosterone, TSH, and TNF‐A Imbalances Caused by MAFLD

**FIGURE 4. Analysis of markers of oxidative status in the testicular tissue:**
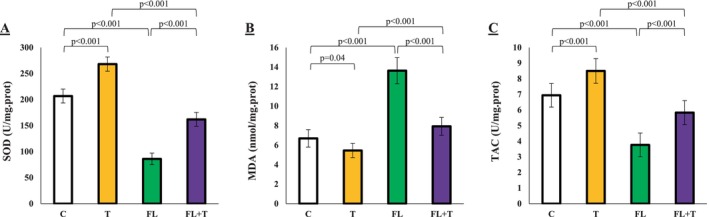
Effect of endurance training on markers of oxidative status in the testicular tissue. Data are presented as means ± standard deviations (average of 3 trials). C, control; FL + T, Fatty liver + endurance training; FL, fatty liver; T, endurance training.

The blood analysis of thyroid hormones in this study revealed a significant decrease in serum testosterone levels, along with a notable increase in TSH and TNF‐a levels in the MAFLD group (*p* < 0.001). However, after eight weeks of endurance training, the levels of testosterone were significantly elevated (*p* < 0.001), while TSH and TNF‐a levels were significantly reduced (*p* < 0.001). These findings suggest endurance training reduces inflammatory complications, reproductive hormonal disorders, and thyroid hormone imbalances caused by metabolic‐associated fatty liver disease (MAFLD) (Figure 4).

### 3.3 Endurance Training Alleviates Oxidative Status Disturbances Caused by MAFLD

The blood analysis of markers of oxidative status in this study revealed a significant decrease in SOD and TAC levels, along with a notable increase in MDA levels in the MAFLD group (*p* < 0.001). However, after eight weeks of endurance training, the levels of SOD were significantly elevated (*p* < 0.001), while MDA levels were significantly reduced (*p* < 0.001). These results suggest that MAFLD induces markers of oxidative status imbalances, and endurance exercise plays a beneficial role in correcting this imbalance by upregulating SOD and TAC and reducing MDA levels (Figure 4).

### 3.4 Endurance Training Mitigates MAFLD‐Induced ER Stress

**FIGURE 5:**
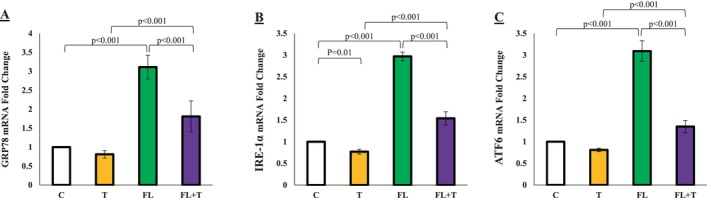
The effect of MAFLD with or without endurance training on the expression of GRP78, IRE‐1α, and AFT6 in the controls and experimental groups. Data are presented as means±standard deviations (average of 3 trials). C, control; FL + T, Fatty liver + endurance training; FL, fatty liver; T, endurance training.

The study findings revealed that MAFLD induction led to a significant upregulation of GRP78, IRE‐1α, and AFT6 gene expression in MAFLD‐affected rats compared to the control group (*p* < 0.001). However, the expression levels of these genes were markedly reduced in the FL + T group compared to the FL group (*p* < 0.001). These results indicate that endurance training mitigates the upregulation of GRP78, IRE‐1α, and AFT6, potentially alleviating endoplasmic reticulum (ER) stress induced by MAFLD. Thus, endurance training appears to exert a protective effect on the testicular tissue of MAFLD‐affected rats by counteracting ER stress (Figure 5).

## 4 Discussion

MAFLD impacts nearly one‐third of the global population, with its development influenced by factors such as genetic predisposition, sedentary behavior, metabolic sexual dimorphism, and dietary habits [51, 52, 53]. Recent studies have established a connection between male infertility and systemic conditions, including MAFLD, cancer, cardiovascular diseases, and autoimmune disorders [7, 24]. Early detection of MAFLD, coupled with timely intervention, can significantly mitigate infertility‐related complications [54]. Among lifestyle modifications, exercise interventions are widely recognized for their ability to reduce risk factors and hinder the progression of MAFLD [55]. This study explored the effect of endurance training on testicular function in a rat model of MAFLD. To our knowledge, this is the first investigation to elucidate the mechanistic role of MAFLD in causing reproductive complications, with a specific focus on the GRP78/IRE‐1α/ATF6 signaling pathway in testicular tissue. Our findings demonstrate that endurance training effectively regulates the expression of endoplasmic reticulum stress pathways, underscoring its protective role in alleviating reproductive dysfunction associated with MAFLD.

MAFLD has been increasingly recognized as a contributing factor to male infertility [7]. It is closely associated with hypogonadism, marked by decreased testosterone levels, which disrupt spermatogenesis and reduce sperm quality [56, 57]. It has also been determined that SHBG is lower in men and women with NAFLD than in people without NAFLD [58]. Several recent studies have found low levels of sex hormone‐binding globulin (SHBG), a protein produced by the liver that transports testosterone and regulates its bioavailability at the tissue level, in obese individuals, men with type 2 diabetes, and men with NAFLD [8]. Furthermore, systemic inflammation and oxidative stress induced by MAFLD exacerbate testicular dysfunction. Recent studies indicate that MAFLD may serve as an early biomarker for testosterone deficiency in aging men without metabolic syndrome [13]. Consistent with these findings, our study revealed that MAFLD reduces sex hormone levels, including testosterone. Notably, previous research reported significantly lower follicle‐stimulating hormone (FSH) levels in men with MAFLD compared to those without the condition [59]. On the other hand, endurance exercise has demonstrated the potential to restore sex hormone levels by ameliorating MAFLD in animal models [60]. In this context, prior studies have shown that regular physical activity can reduce body mass index and alleviate obesity‐related complications [61, 62], which may explain its positive effect on MAFLD‐associated infertility and sexual dysfunction. Moderate‐intensity exercise, in particular, has been shown to enhance antioxidant defenses and prevent cell apoptosis, further reinforcing its protective role [47]. On the other hand, Xu et al. showed that moderate‐intensity and high‐intensity exercise protect against HFD‐induced oxidative stress and apoptosis in testicular tissues, but high‐intensity exercise has a better effect [60]. Research by Ghareghani et al. [63] highlights the lipid‐regulating effects of endurance training through autophagy, while other studies confirm its efficacy in mitigating MAFLD in animal models. Collectively, these findings emphasize the importance of lifestyle interventions, especially regular exercise, in improving liver function and male reproductive function; however, more research is still needed to determine the right intensity of training.

Recently, it has been widely accepted that the spleen is likely to be involved in any inflammatory process, such as obesity and NAFLD, by secreting specific mediators [64]. Studies have shown larger spleen volumes in individuals with NAFLD and type 2 diabetes compared to controls, suggesting an association between spleen volume and NAFLD in the early stages of the disease, possibly due to an initial increase in portal venous pressure [65]. It has also been found that testosterone may directly and/or indirectly affect the spleen. For example, the spleen exhibits a relatively high binding capacity for testosterone, and the number of cells in the spleen has been reported to decrease with testosterone treatment [66]. On the other hand, testosterone deficiency appears to exacerbate inflammation. Low testosterone levels are associated with increased cardiovascular risk factors [67], mortality [68], diabetes [69, 70], and specifically with increased levels of TNF‐α or other inflammatory mediators [71, 72]. These findings, together with the observation of a reduction in cytokines by testosterone, suggest that testosterone may have potent anti‐inflammatory effects [64]. Thus, increasing testosterone levels after exercise could be a therapeutic strategy to reduce the inflammatory effects of a dysfunction in the spleen‐liver axis and thereby improve its associated diseases. These results represent a major experimental step forward in defining the tissues and/or cells that mediate the sensitization or non‐healing effects of testosterone.

MAFLD has also been implicated in disruptions of thyroid hormone homeostasis, with evidence suggesting a bidirectional relationship between thyroid hormones and the risk of developing MAFLD [14]. Thyroid hormones play a critical role in regulating lipid metabolism, and their dysregulation can promote hepatic lipid accumulation, triggering inflammation and contributing to MAFLD [73]. Our results demonstrate that MAFLD significantly impairs the levels of TSH and TNF‐a. However, endurance exercise effectively normalized thyroid‐stimulating hormone and TNF‐a levels in MAFLD‐induced rats. It is worth noting that the effects of endurance training on thyroid hormones remain contentious, as some studies report no significant changes, while others indicate varying degrees of increase or decrease [74, 75]. Despite this variability, the findings of our study suggest a beneficial regulatory effect of endurance exercise on thyroid hormone balance in the context of MAFLD.

Obesity is closely related to male reproductive dysfunction [76]. Oxidative stress plays an essential role in obesity‐induced reproductive disorders [77, 78]. Obesity can lead to systemic oxidative stress, which induces ROS accumulation [79]. In this study, we found that 8 weeks of endurance training protected against HFD‐induced oxidative stress in testicular tissues; as a result, testicular morphological and functional impairment improved. It has been determined that when the levels of MDA and the lipid peroxidation byproducts were higher than the activity of antioxidant enzymes, testes were vulnerable to oxidative damage [80]. In this regard, a study showed that 8 weeks of swimming training significantly reduced oxidative stress levels and increased antioxidant capacity in the testes of obese mice [81]. In line with these findings, our study found that endurance training decreased the activities of MDA and restored the activities of SOD and TAC in the testes of HFD‐fed rats.

Endoplasmic reticulum (ER) stress, a cellular condition arising from improper protein folding and modification, plays a pivotal role in the onset and progression of MAFLD [82]. Persistent ER stress can lead to cell death and has been linked to azoospermia and impaired sperm production through its effects on spermatogonia in the testes [27, 54]. Our findings reveal a marked upregulation of GRP78‐IRE‐1α‐ATF6 gene expression in the testicular tissue of MAFLD‐induced rats, indicating a pronounced ER stress response triggered by a high‐fat diet. This study is the first to demonstrate that MAFLD‐induced testicular dysfunction may be mediated by activation of the GRP78‐IRE‐1α‐ATF6 pathway, resulting in apoptosis of Leydig and Sertoli cells and impairing testosterone production and spermatogenesis. Conversely, endurance training significantly modulated the expression of GRP78‐IRE‐1α‐ATF6 genes, thereby mitigating ER stress. Previous research has shown that endurance exercise induces the unfolded protein response via ER stress, often driven by elevated reactive oxygen species (ROS) during skeletal muscle contraction [83]. However, the endurance training protocol employed in our study was deliberately designed to be non‐stressful and reflective of human lifestyle habits. This controlled regimen allowed for sustained physical activity, replicating the intensity and duration typical of human endurance training programs. Thus, the protocol effectively addressed testicular damage‐related complications associated with MAFLD, underscoring its potential translational value in clinical practice.

## 5 Conclusion

This study demonstrates that endurance training effectively reduces obesity in MAFLD‐affected rats while modulating the ER stress signaling pathway in the testes. This intervention also led to the regulation of the levels of key variables, including TSH, testosterone, TAC, MDA, and SOD. These results highlight the critical role of modulated ER stress as a mechanism through which endurance exercise mitigates MAFLD‐induced testicular dysfunction. Collectively, the evidence positions endurance training as a promising therapeutic strategy for addressing MAFLD and its associated reproductive complications. Compared to pharmacological treatments, exercise offers a cost‐effective, low‐risk alternative that is accessible to both affected individuals and at‐risk populations. However, future research should aim to elucidate the detailed molecular pathways that underpin the protective effects of endurance training and other exercise modalities on MAFLD‐related reproductive dysfunction. Long‐term clinical trials are also essential to evaluate the translational relevance of these findings in humans, particularly to establish the most effective exercise regimens for enhancing male fertility in individuals with MAFLD.

## Author Contributions

V.K. and N.T. conceptualized the study and developed the research plan. V.K. conducted animal experimentation. V.K. and N.T. designed and conducted biochemical and molecular analysis. V.K. performed data analysis. V.K. and N.T. wrote the initial manuscript draft. V.K. provided comprehensive editing to finalize the manuscript. V.K. supervised the project. All authors provided critical feedback and played a role in shaping the research, analysis, and manuscript preparation.

## Ethics Statement

All procedures were performed under the guidelines established by the Animal Care and Research Ethics Committee of Kermanshah University of Medical Sciences (IR.KUMS.AEC.1402.047).

## Conflicts of Interest

The authors declare no conflicts of interest.

## Data Availability

The data that support the findings of this study are available from the corresponding author upon reasonable request.
